# Age-Related Macular Degeneration in the Aspect of Chronic Low-Grade Inflammation (Pathophysiological ParaInflammation)

**DOI:** 10.1155/2014/930671

**Published:** 2014-08-19

**Authors:** Małgorzata Nita, Andrzej Grzybowski, Francisco J. Ascaso, Valentín Huerva

**Affiliations:** ^1^Domestic and Specialized Medicine Centre “Dilmed”, Bohaterów Monte Cassino 3, 40-231 Katowice, Poland; ^2^Department of Ophthalmology, University of Warmia and Mazury, Al. Warszawska 30, 10-082 Olsztyn, Poland; ^3^Department of Ophthalmology, Jozef Strus Poznan City Hospital, 3 Szwajcarska Street 61-285 Poznan, Poland; ^4^Department of Ophthalmology, “Lozano Blesa” University Clinic Hospital, 50009 Zaragoza, Spain; ^5^Aragon Health Sciences Institute, 50009 Zaragoza, Spain; ^6^Department of Ophthalmology, University Hospital Arnau de Vilanova, 25198 Lleida, Spain; ^7^IRB-Lleida, 25198 Lleida, Spain

## Abstract

The products of oxidative stress trigger chronic low-grade inflammation (pathophysiological parainflammation) process in AMD patients. In early AMD, soft drusen contain many mediators of chronic low-grade inflammation such as C-reactive protein, adducts of the carboxyethylpyrrole protein, immunoglobulins, and acute phase molecules, as well as the complement-related proteins C3a, C5a, C5, C5b-9, CFH, CD35, and CD46. The complement system, mainly alternative pathway, mediates chronic autologous pathophysiological parainflammation in dry and exudative AMD, especially in the Y402H gene polymorphism, which causes hypofunction/lack of the protective complement factor H (CFH) and facilitates chronic inflammation mediated by C-reactive protein (CRP). Microglial activation induces photoreceptor cells injury and leads to the development of dry AMD. Many autoantibodies (antibodies against alpha beta crystallin, alpha-actinin, amyloid, C1q, chondroitin, collagen I, collagen III, collagen IV, elastin, fibronectin, heparan sulfate, histone H2A, histone H2B, hyaluronic acid, laminin, proteoglycan, vimentin, vitronectin, and aldolase C and pyruvate kinase M2) and overexpression of Fcc receptors play role in immune-mediated inflammation in AMD patients and in animal model. Macrophages infiltration of retinal/choroidal interface acts as protective factor in early AMD (M2 phenotype macrophages); however it acts as proinflammatory and proangiogenic factor in advanced AMD (M1 and M2 phenotype macrophages).

## 1. Introduction 

Age-related macular degeneration (AMD) is the leading cause of permanent, irreversible, central blindness (scotoma in the central visual field makes impossible the following: reading and writing, stereoscopic vision, and recognition of colours and details) in patients over the age of 50 in industrialized European and North American countries. Soft drusen and/or pigmentary abnormalities are clinically visible symptoms of early AMD. Late (or advanced) AMD occurs in two distinct forms, and visual loss is caused by the “geographic atrophic” death of photoreceptors and retinal pigment epithelium (RPE) cells, the so-called dry AMD, GA/AMD, or by formation of the choroidal neovascular membrane (CNV), as a result of pathological angiogenesis, the so-called exudative or wet AMD, CNV/AMD [1–3].

Hypoxia, as well as hyperactivity of the complement system, together with the inflammatory process, leads to disturbances in the pro/antiangiogenic balance and RPE cells overexpression of proangiogenic vascular endothelial growth factor (VEGF), which plays a key role in the pathogenesis of CNV/AMD [1, 4]. AMD is a complex degenerative and progressive disease of multifactorial etiology, and advanced age (and its related physiological cell apoptosis and tissue involution) and genetic predisposition are the strongest risk factors; however, important are also other factors such as sex and environmental influences (smoking cigarettes, heart and vascular disorders, hypertension, dyslipidemia/hypercholesterolemia, diabetes, obesity, improper diet, sedentary lifestyle, and phototoxic exposure) [1–3, 5, 6]. Despite intensive researches, the pathomechanism of AMD has not yet been fully recognized, since the pathogenesis of AMD is likely to involve the disruption of multiple physiological pathways [7]; however, according to many authors, inflammation and especially the complement system play key role in the etiopathogenesis of AMD. Inflammation is involved in both late forms of AMD, that is, in GA/AMD and CNV/AMD [8–13].

## 2. Chronic Low-Grade Inflammation (Pathophysiological Parainflammation) Model of AMD Etiopathogenesis 

### 2.1. General Characteristics of Normal/Pathophysiological Parainflammation Process

The term “parainflammation,” first proposed by Medzhitov, means tissue adaptive response to noxious stress or malfunction and has characteristics that are intermediate between basal and inflammatory states. It is not a classic form of inflammation caused by exogenous tissue injury or foreign antigen (infection). Parainflammation is a local low-grade/subclinical immune reaction, caused by endogenous inducers (they may not be detectable by using common inflammatory biomarkers), to adapt tissue to harmful environment and to maintain their adequate functionality. In other words, parainflammation is a state between healthy homeostasis and chronic inflammation [14]. However, if the noxious stress or malfunction is prolonged and not removed, then parainflammation progresses into a chronic condition, with classic well known and defined inflammatory pathways, and leads to tissue damage [14]. Oxidative stress is considered by many authors to be the main initial determinant for parainflammatory responses [7–9]. Parainflammation means the activation of locally resident immune cells in the first instance and the induction of complement system, mainly its alternative pathway, in chronic conditions [7–9].

Normal, adaptive parainflammation exists in the aging retina under physiologic conditions, as a protective response against low-grade noxious waste products accumulated in Bruch's membrane, and allows maintenance of retinal homeostasis [8, 9]. However, prolonged period of retina exposition to stress and/or malfunction leads to chronic unbalanced and uncontrolled parainflammation process, due to either prolonged and aggressive tissue damage (caused by extreme aging or unhealthy life style) or decreased immune system ability to repair tissue (caused by premature aging or genetic susceptibility) [9]. Dysregulated normal parainflammation evolves into a chronic inflammatory response [9]. Such “chronic abnormal inflammatory process” [15], or failure “chronic para-inflammation” [7, 9] or “pathophysiologic para-inflammatory response” [8], leads to chronic detrimental inflammatory reactions and immunologic events [9, 15], promotes tissue damage, and contributes to the initiation of AMD [9, 16, 17], as well as playing an important role in AMD progression [9, 15].

According to many authors, AMD is not classic (regular) inflammation but is a condition of “chronic low-grade/subclinical degree of inflammation (pathophysiologic parainflammation)” [8, 9], which plays role in every AMD form [18]. In the course of AMD, chronic, pathophysiological parainflammatory process occurs in the macula and in paracentral retinochoroidal tissues and is related to the anatomy-activity complex consisting of the outer photoreceptors, RPE cells, Bruch's membrane, and choriocapillaris [7–9].

Parainflammation is characteristic for tissues functionally dependent on nonproliferative cells and characterized by very high metabolism [8]. Photoreceptors, RPE, and ganglion cells are terminally differentiated (postmitotic) retinal cells, with limited or lack of ability to regenerate [9]. Neuroretina is a highly metabolic tissue, sensitive to noxious factors, and constantly exposed to light stimulation, which generates large amounts of oxidized materials [9]. The major causes of tissue stress resulting in local triggers for parainflammation are oxidative stress and oxidative species, that is, reactive oxygen species (ROS) and reactive nitrogen species (RNS), oxidized lipoproteins, advanced glycation endproducts (AGEs), and apoptotic cells [7–9].

### 2.2. Characterization of Main Normal/Pathophysiological Parainflammation Triggers

Reactive oxygen and nitrogen species are generated during normal physiological processes. Oxidative stress occurs when the production of ROS and RNS accelerates, or when the mechanisms involved in their rapid removal are impaired [9]. In the retina, the inner segments of photoreceptors and RPE cells, both rich in mitochondria organelles, are the main source of oxidative and nitrative species in the pathophysiological conditions [9]. Sunlight exposure, mitochondrial respiration, phagocytosis of the outer photoreceptors segments by RPE cells, photosensitization of protoporphyrin, and lipofuscin phototoxicity, as well as unregulated choroidal blood flow, connected with increased fluctuations of tissue oxygen concentration, predispose the aging macula to oxidative stress and elevated mitochondrial ROS and RNS creation [19, 20]. ROS impair cells function by reacting with nucleic acids, proteins, and lipids [21]; they also induce production of proinflammatory cytokine [22] and angiogenic signals [23] and may lead to apoptosis of photoreceptors, RPE cells, and retinal ganglion cells in the aging retina [24]. RNS trigger damage of proteins and lipids [25], cause nitrosative stress in the aging retina [9], and may induce apoptotic death of photoreceptors [26].

Oxidative and nitrative species trigger parainflammation process directly, since oxidized or nitrated lipids and proteins stimulate inflammatory responses, as well as indirectly activating the tissue resident macrophages to express proinflammatory mediators [9]. Moreover, RON may alter vascular permeability and breakdown of blood-retina barrier [27], which promote release of proinflammatory factors [9].

### 2.3. Oxidized Lipoproteins

The retina, particularly the photoreceptor layer rich in unsaturated lipoproteins, is readily susceptible to oxidation by ROS. Lipid oxidation increases with the age and occurring oxidized low-density lipoproteins (OxLDL) are particularly relevant to retinal parainflammation [9]. OxLDL bind to RPE cells and retinal microglia via scavenger receptors, that is, via CD36, LDL-R, and newly described lectin-like oxidized lipoprotein receptor 1 (LOX-1) detected in RPE cells and in microglia [28, 29], as well as via the CD68 receptor detected in microglia [30]. OxLDL promote parainflammation activating lipid-laden monocytes and macrophages to secrete cytokines (IL-8), growth factors (TNF-alpha), and matrix metalloproteinases. Immune complexes consisting of OxLDL and antibody activate also complement system pathway [9].

The term advanced glycation endproducts(AGEs) is a broad term which includes various proteins and lipids undergoing chemical reactions after exposure to sugars; their physiological serum level is in the range of 2–10 Lg/mL and increases with the age [31]. AGEs promote inflammation in many general age-related diseases such as Alzheimer's disease, atherosclerosis, diabetes, osteoarthritis [32–34], and AMD [35]. AGEs represent risk factor of AMD development [36].

AGEs are a constituent of drusen [37] and accumulate in Bruch's membrane in the course of AMD [38]. They stimulate RPE cells to secrete different anti/proinflammatory factors, which trigger parainflammation state in RPE cells, that is, short-term adaptive RPE cell reaction on AGE stimulation [39]; however chronic RPE exposure to AGE favors deregulation of RPE function and leads to photoreceptors and RPE cells degeneration and atrophy [38].

According to Lin et al., anti-inflammatory cytokines including IL10, IL1ra, and IL9 were overexpressed and proinflammatory cytokines including IL4, IL15, and IFN-*γ* were overexpressed, while other proinflammatory cytokines including IL8, MCP1, and IP10 were underexpressed with AGE stimulation of human cultured RPE cells [39]. Upregulated were also chemokine CXCL11 and viperin RSAD2 [39]. Strongly immunoreactive CXCL11 protein, associated with drusen, mediates chemotaxis between cells in inflammatory response.

Multifactorial antiviral protein RSAD2 is also localized in drusen and outer retina, mediating inflammatory response, abnormal lipid accumulation, and atherosclerosis [39].

Extracellular receptors which bind AGEs on RPE cells include toll-like receptors (TLRs), receptors for advanced glycation endproducts (RAGEs), and advanced glycation endproducts receptors (AGERs). TLRs and RAGEs activate inflammatory signals within the cells, and AGERs are important inhibitors of these signals [39].

According to Lin et al., all the above cytokines are novel agent (genes) associated with AMD pathogenesis and candidate marker of inflammatory events in the outer retina, and modulating their activity associated with specific pathways (NF-jB and JAK-STAT pathways) may be new therapeutic strategy to limit RPE dysfunction and photoreceptor loss in the course of AMD [39].

AGEs also induce secretion of proangiogenic cytokines by RPE cells (upregulation of VEGF in RPE cells after stimulation by AGEs) [40]. AGEs are endocytosis and removed by macrophages [41]. The failure in macrophage recruitment (see below) may lead to increased RPE exposure with AGEs and damage retinal tissue in the pathological angiogenesis process [39].

### 2.4. Soft Drusen as Clinical Symptom of Chronic, Pathophysiological Parainflammation Process in the Retinal/Choroidal Interface

In the aging retina, the cumulative effect of oxidative stress, endoplasmic reticulum stress, and accumulation of RPE byproducts lead to deposition of glycoproteins, lipids, and cellular debris, as well as creating clinically invisible basal deposits, that is, basal laminar deposits (BLamD) and basal linear deposits (BLinD). BLamD are accumulated between the cytoplasmic and the basement membrane of RPE cells, and BLinD are stored in the extracellular matrix, between the RPE basement membrane and the internal collagen layer of the Bruch's membrane [42–44]. Basal deposits deepen the lipofuscinogenesis process in RPE cells, which means accumulation of lipofuscin (age pigment) developed from fully or partially digested outer segments of photoreceptors, as a result of the gradual failure of RPE liposomal enzymes [42–44]. BLamD and BLinD with diameters of over 25–30 *μ*m turn into clinically visible macular and perimacular soft drusen (SD), the hallmark of early AMD, and a risk factor for developing advanced forms of AMD during the next 5 to 10 years [45, 46], that is, GA/AMD or CNV/AMD [47–49].

Soft drusen are not only the side effect of dysfunction and degeneration of RPE cells [5, 43, 44, 46], but they indicate also a chronic, pathophysiological parainflammatory process in extracellular matrix. SD are the earliest diagnosable byproducts of local detrimental parainflammatory process and act as foci of chronic abnormal inflammation in Bruch's membrane [5, 12, 13, 37, 50]. They induceactive, nonspecific inflammatory cells reactions [46, 51] and are biomarkers of immune-mediated processes in RPE/Bruch's membrane interface [52–54].

Inflammatory etiopathogenesis of human soft drusen confirms their content, which has been recognized during the course of numerous histochemical studies. Soft drusen are composed of phospholipids, glycolipids, cholesterol, saturated and unsaturated fatty acids, carbohydrates, and approximately 130 various kinds of proteins, among which are apolipoproteins, vitronectin, clusterin, ubiquitin, fibronectin, integrins, extracellular matrix metalloproteinases, and their inhibitors [13, 37, 55]. Soft drusen contain also numerous mediators of inflammation such as C-reactive protein (CRP) [13, 56], adducts of the carboxyethylpyrrole protein (CEP) [57], immunoglobulins (IgG) [58], acute phase molecules (vitronectin, amyloid P, and fibrinogen), and apolipoproteins [59] as well as many complement-related proteins such as complement components (C3a and C5a proteins) [60], terminal complement complex (C5 and C5b-9 proteins) [54, 56], fluid-phase complement regulators (complement factor H-CFH, vitronectin, and clusterin) [61], and membrane-bound complement inhibitors (complement receptor1-CR1, also called CD35 and membrane cofactor protein-MCP, also called CD46) [61, 62].

Many complement-related proteins such as complement system activation products (C3a, C5, and C5b-9 proteins), complement activators (CFH, CD35, and CD46 proteins), and complement regulators have shown not only in drusen, but also in the capillary pillars of the choroid and within surgically removed choroidal neovascular membranes [37, 54, 62, 63], as well as in the human vitreous body [4].

### 2.5. Chronic Autologous Pathophysiological Parainflammation Mediated by Complement System in Retinal/Choroidal Interface

The complement system is a specific biochemical cascade and an integral element of inborn (not specific) immunological response, which supports (complements) the host antibodies ability to destroy pathogens, promotes removal of apoptotic cells and immune complexes, and enhances an individual adaptive immune response [64]. Its deregulation or dysfunction leads to various autologous diseases [65, 66] and is implicated in the etiopathogenesis and progression of AMD [63, 65]. The complement system consists of more than 40 proteins and regulators synthesized by the liver and circulating in the blood as inactive precursors or proproteins. They act in three distinct complement pathways activated by specific trigger. The classical pathway is triggered by antigen-antibody complexes, the alternative pathway is activated by binding to membranes of pathogen or host cell, and lectin pathway is triggered by binding serum lectin to mannose residues (polysaccharides) on microbial surfaces. The alternative and lectin pathways are called nonspecific immune response pathways, since they do not require the presence of a typical pathogen and act without the presence of antibodies.

Activation of these pathways results in a proinflammatory response including generation of cell-killing terminal complement complex (TCC), also called the membrane-attack complex (MAC), which mediate aggressor's cells lysis, after the spontaneous hydrolysis of the C3 factor (classic pathway) or the C3b factor (alternative pathway), release of chemokines to attract inflammatory cells to the site of damage, and enhancement of capillary permeability [63, 67, 68].

The complement system is continuously activated at low levels in the normal eye, but intraocular complement regulators (CFH, CD35, and CD46 proteins) tightly regulate its spontaneous activation to maintain activity at a level that promotes elimination of pathogens and waste products without damaging healthy tissue, and leukocytes, lymphocytes, and macrophages additionally support the complement system in cleaning the extracellular matrix [67, 68]. With the age, the longstanding photooxidation process and oxidized albuminous components of lipofuscin deregulate complement system and play pivotal role in the activation of classic and alternative pathways, which leads to the chronic immune inflammatory process in the macula in the course of AMD [37, 57, 69].

Oxidized components of lipofuscin, that is, isolevuglandin, bis-retinoid pyrimidine A2E, and carboxyethylpyrrole protein (CEP), and its adducts (generated in blood as a result of CEP conjugation with circulating plasma albumin) are present in lipofuscin of RPE cells, in soft drusen, and in systemic circulation [37, 57, 69]. These strongly immunogenic factors constitute a target for the immune system and stimulate the production of antibodies (CEP protein) and endogenic, autoantibodies (CEP protein adducts) [37, 57], which lead to autologous immune-mediated inflammation, strong attack of both the drusen material and its own RPE cells, cells apoptosis, and finally maculae damage [63, 67, 68].

In AMD, the complement system activation is not only an effect, but also an evidence of local, strong, and chronic deregulation of immune system in Bruch's membrane and complement system pathognomonic role has been confirmed in both: dry AMD [13] and wet-AMD [70–73].

### 2.6. The Influence of Gene Polymorphism on Pathophysiological Parainflammation Activity

Autologous inflammation mediated by overactive alternative pathway is especially strong in AMD patients with the Y402H gene polymorphism, since it causes hypofunction or lack of the protective complement factor H (CFH). CFH is a serum suppressor of alternative complement activity in plasma, in host cells and tissue, and in sites of tissue inflammation. CFH maintains local homeostasis and protects macula against deep disintegration [7]. CFH is produced locally in the eye and accumulated in the interphotoreceptor matrix, in RPE cells and the sub-RPE space, in soft drusen, and in the choroids [61, 74]. CFH binds to antigen epitopes, the part of an antigen recognized by antibodies, B cells, or T cells, and protects against oxidative stress [75]. CFH is involved in inhibiting the inflammatory response mediated via C3b (the alternative pathway of complement) both by acting as a cofactor for cleavage of C3b to its inactive form C3bi and by weakening the active complex which is formed between C3b and factor B [7]. CFH slows down the cascade of reactions connected with an increase in TCC, especially within the range of the alternative pathway [61, 76, 77], and the mutation in CFH (Tyr402His) reduces the ability of CFH to regulate the alternative pathway permitting it to run uncontrolled [61, 68, 74].

The CFH-Y402H gene polymorphism reduces also the CFH affinity for C-reactive protein (CRP) and results in the high levels of unbound CRP, which facilitate chronic inflammation [78, 79]. C-reactive protein (CRP) is a nonspecific serum biomarker for acute phase of subclinical inflammation [80]. CRP induces innate immune response to infection and/or tissue injury, since CRP activates complement directly, via alternative pathway, and indirectly, via CRP-CFH interaction, which improve CFH ability to inhibit complement. CRP may also enhance phagocytosis of macrophages, which express CRP receptor [80]. There is a significant inverse correlation between the CRP and CFH levels in patients with advanced AMD and CFH gene polymorphism, which means that high level of CRP and insufficient level of CFH at RPE/Bruch's membrane/choroid complex may lead to uncontrolled complement activation and macula damage [78]. Moreover, CRP serum level >3 mg/L is related to a double AMD risk in comparison with CRP concentration <1 mg/L [81], and elevated serum CRP level together with homozygous CFH-Y402H polymorphism leads to 19.3 risk ratio of AMD and to 6.8 risk ratio of AMD progression [82]. According to what is above some authors state that CRP has a direct responsibility in macular damage via complement mediated mechanisms, especially in cases with CFH-Y402H gene polymorphism, and CRP is a risk factor for developing AMD [83, 84]. However, the full mechanism of CRP influence on AMD is not understood [7], and some authors contradict the relation between CRP and AMD [85, 86].

Apart from complement factor H, also other serum suppressors of the complement system activity are important, including the family of factor H-like proteins 1–5, C4-binding proteins, and surface protein such as membrane decay-accelerating factor CD55, membrane cofactor protein CD46, and receptors of the complement system CR1, CR2, and CR3 [64, 87].

The strong support for the immunoinflammatory model of AMD pathogenesis is the highly significant correlations between AMD and polymorphism of genes encoding proteins directly involved in the complement alternative pathway activity. Polymorphism of genes encoding complement factor H, complement component 3, complement factor I, and CFH-related genes-2-4-5 increases AMD risk, and polymorphism of genes encoding complement factor B, complement component 2, and CFH-related genes-1-3 reduces risk of AMD development [88].

### 2.7. Chronic Pathophysiological Parainflammation Associated with Microglial Activation in Retinal/Choroidal Interface

Microglial cells are specialized tissue macrophages resident in the retina and are responsible for detecting noxious stimuli in local microenvironment [89]. Microglial activation is triggered by different endogenous factors such as oxidized protein and lipid, as well as AGEs [90]. In the aging retina, microglial activation is associated with the breaking down of the outer blood retinal barrier [9]. Activated microglial cells (large cell body and short dendrites are morphological signs of activation) migrate from the neuroretina into the subretinal space, that is, into potential space between outer segment of photoreceptors and the apical surface of the RPE cells in order to digest (phagocyte) their accumulated deposits in Bruch's membrane [30, 91, 92]. Microglial activation and coexisting parainflammation process allow the maintenance of local retinal homeostasis and is a form of protective response against low-grade noxious elements in the aging retina [9, 93]. However, in cases of prolonged stress and/or increased immunological responses or loss of mechanisms of their sufficient control, chronic microglial activation and chronic parainflammation state induce proapoptotic events and cause photoreceptor cells injury [94]. Parainflammation connected with microglial activation leads to the development of dry AMD [95].

### 2.8. Chronic Pathophysiological Parainflammation Associated with the Activity of Autoantibodies and Immune Complexes in Retinal/Choroidal Interface and in the Retina

Patients with CNV/AMD have autoantibodies to various antigens such as alpha beta crystallin, alpha-actinin, amyloid, C1q, chondroitin, collagen I, collagen III, collagen IV, elastin, fibronectin, heparan sulfate, histone H2A, histone H2B, hyaluronic acid, laminin, proteoglycan, vimentin, and vitronectin systemically expressed [96]. Many of the above proteins are the building elements of extracellular matrix, that is, the 2–4 *μ*m thick Bruch membrane [97], which acts as a frame for the metabolically active RPE cells and a physical barrier for their passage and that of the endothelial vessels and regulates the diffusion of nutrient molecules between the retina and the choroid [97]. Collagens type I and type III (fibrillar), elastin, fibronectin, and proteoglycans (mainly heparan sulfate proteoglycans) are the main structural components of internal/outer collagenous and elastic layer of Bruch's membrane, and collagen type IV (nonfibrillar), laminins, and proteoglycans (mainly heparan sulfate proteoglycans) are the main structural components of RPE and choriocapillaris basement membranes [5]. The presence of such numerous autoantibodies against main structural elements of Bruch's membrane indicates strong immunological process in extracellular matrix, which may facilitate penetration of neovascular membrane through choroidal/retinal interface under neuroretina in exudative AMD. Sera of patients with AMD indicate also the presence of other autoantibodies, that is, autoantibodies against aldolase C (ALDOC) and pyruvate kinase M2 (PKM2). ALDOC and PKM2 are enzymes associated with glucose metabolism and are specific for dry and wet AMD. Autoantibodies against ALDOC/PKM2 indicate activity disorders stimulated by the immunological inflammatory condition, which in turn may deepen the structural changes in the Bruch membrane. According to Morohoshi anti-PKM2 IgG should serve as a biomarker for diagnosis and prognosis of AMD [96].

Antibodies mediate inflammation by activation of the classic pathway of complement cascade (when they bind their cognate antigen and form immune complexes) or by binding Fcc receptors (FccRs) on effector cells such as macrophages [98]. The FccRI, FccRIIa, FccRIIc, FccRIIIa, and FccRIIIb are the activating receptors, and FccRIIb is the inhibitory receptor in humans. Activated/inhibited FccRs activate/inhibit function of effector cells, that is, their capability of phagocytosis, cytokine production, and degranulation [99]. Immune complexes produce neuroinflammation in the retina. In early AMD, formation of immune complexes in the choriocapillaris was accompanied with upregulation of Fcc receptors. Large number of leukocytes expressing Fcc receptors was observed also in exudative AMD. It implicates a possible role for antibody-mediated inflammation in early and advanced AMD largely dependent on the presence and activity of Fcc receptors [100].

### 2.9. Chronic Pathophysiological Parainflammation Associated with Choroidal Macrophages Infiltration

Leukocytes promote endothelial cell injury and migrate through the endothelium to other tissues. They may infiltrate the retina and injure the macula [101]. It is favored by higher expression of intercellular adhesion molecule-1 (ICAM-1) in the macula, as compared to the peripheral retina [102]. Elevated white blood cell counts correlate with the presence of soft drusen [103] and CNV/AMD [104], as well as possibly with GA/AMD [103], which indicates the relationship of systemic circulating, nonspecific chronic inflammatory cells with the pathogenesis of early and advanced AMD.

Monocytes after migration from bloodstream into the subendothelial space differentiate into mature tissue resident macrophages. Their recruitment into inflamed tissues mediates chemokines (signaling proteins) released by damaged or infected cells [105]. Locally produced monocyte chemoattractant protein (MCP)-1 (CCL2) is one of the key chemokines which regulates migration and infiltration of monocyte. MCP-1/CCL2 interact with the chemokine receptors type 1 and 2 (CX3CR1 and CCR2, resp.) [106, 107]. Macrophages induce granulomatous inflammatory reactions in Bruch's membrane [46, 51, 108]. They were found in the choroids of AMD patients [109], in degraded areas of Bruch's membrane [110, 111], and in surgically excised CNV membranes [112].

Macrophages represent a broad phenotypes spectrum and play dual role in AMD pathogenesis. Anti-inflammatory macrophages maintain balance of waste products in the aging Bruch membrane and remove SD and other deposits. Proinflammatory macrophages maintain parainflammation state of AMD lesions and promote the development of late stages of disease [113, 114].

M1 phenotype macrophages have proinflammatory potency and induce tissue damage [113]. M2 phenotype macrophages are less inflammatory. In early AMD, M2 macrophages act mainly as scavenger and anti-inflammatory factor; since they digest proinflammatory agents such as AGEs, complement complexes, oxidized membrane, and dead cells, removal of apoptotic cell debris limits inflammation through inhibiting the production of inflammatory cytokines [113, 114]. Failure in recruitment M2 macrophages, caused by inhibition of receptor CCR2, essential for scavenging deposits [115] leads to increased RPE cells exposure to AGEs, which may stimulate RPE cells to secrete proangiogenic cytokines, including VEGF [40]. In late AMD, M2 macrophages represent proangiogenic activity and facilitate CNV formation, since they may express proangiogenic cytokines (including VEGF), growth factors, and reactive oxygen species negatively affecting the RPE and choriocapillaris, and they may promote proteolysis of Bruch's membrane as well. They also serve as scaffold for CNV development [113, 114, 116].

## 3. Summary

Etiopathogenesis of AMD involves the disruption of many physiological pathways, but chronic pathophysiological parainflammation plays a key role in AMD development. Normal, adaptive parainflammation process exists in the aging retina under physiologic conditions, but unbalanced and uncontrolled parainflammation leads to detrimental chronic inflammatory response and causes early and advanced AMD forms. Chronic pathophysiological parainflammation is mediated by many factors and stimulated by complement system, especially its alternative pathway, which is carried out in Bruch's membrane, and leads to early and advanced, exudative AMD forms. Chronic pathophysiological parainflammation connected with microglial activation carried out also in retinal/choroidal interface leads to advanced atrophicans form of AMD. Chronic pathophysiological parainflammation connected with autoantibodies and formation of immune complexes carried out in Bruch's membrane lead to early and advanced AMD. Chronic pathophysiological parainflammation connected with choroidal macrophages infiltration leads to exudative form of AMD.
